# YOLOBT: a novel ERP bad trial detection network dynamically adjusting based on global signal quality

**DOI:** 10.3389/fnhum.2026.1714086

**Published:** 2026-03-10

**Authors:** Zhaojin Chen, Lijuan Duan, Lei Liu, Xixi Zhao, Changming Wang

**Affiliations:** 1 School of Computer Science, Beijing University of Technology, Beijing, China; 2 Beijing Key Laboratory of Trusted Computing, Beijing, China; 3 National Engineering Laboratory for Key Technologies of Information Security Level Protection, Beijing, China; 4 Xuanwu Hospital, Capital Medical University, Beijing, China; 5 Department of Neurology and Innovation Center for Neurological Disorders, Beijing, China; 6 National Center for Neurological Disorders, Beijing, China; 7 Anding Hospital, Capital Medical University, Beijing, China

**Keywords:** bad trial, deep learning, electroencephalography (EEG), Transformer, YOLOv5

## Abstract

Event-related potentials (ERPs) are time-locked voltage changes in averaged EEG signals reflecting neural responses to specific events. ERPs are extracted from EEG by repeating the same stimulus across multiple trials and averaging the recordings. In ERP studies, artifact-contaminated trials (commonly termed “bad trials”) refer to data segments deemed unsuitable for analysis due to excessive noise or artifacts. The criteria for determining such trials depend on overall data quality: researchers increase artifact tolerance when a subject's data quality is poor to retain statistical power, while applying stricter standards when quality is high to ensure analytical purity and accuracy. Current automated bad trial detection methods rely on static thresholds and fail to replicate the adaptive strategies employed by experts. To address this limitation, we propose YOLOBT, a YOLO-based deep learning framework that mimics expert judgment by integrating global signal quality assessment with dynamic threshold adjustment. By treating EEG signals as visualized waveform images, our approach naturally aligns with expert visual inspection methods while enabling context-aware artifact detection. Our technical contributions include: (1) a Cross-Layer Attention Bottleneck (CLAB) enhancing artifact feature extraction through cross-layer attention mechanisms; (2) a Hierarchical Feature Guidance Module (HFGM) leveraging high-level semantic features to guide low-level feature refinement; and (3) a Global Information Classification Module (GICM) enabling dynamic threshold adjustment based on comprehensive signal quality assessment. Experiments on our manually annotated dataset showed YOLOBT achieved 88.76% precision, 86.89% recall, 92.76% mAP, and 87.82% F1 score, outperforming classical models. Heatmap visualization confirmed the model adaptively adjusts artifact detection strategies based on signal quality, similar to expert judgment processes.

## 1 Introduction

Since its introduction in the early 20th century, Electroencephalography (EEG) (Niedermeyer and da Silva, 2005; Cohen, 2017) has undergone significant advancements. Today, it has become an indispensable tool for studying brain activity in fields such as neuroscience (Zhang et al., 2023), psychology (Wei et al., 2022), and clinical medicine (Porcaro et al., 2023). ERP (Picton et al., 2000) refers to scalp-recorded electrical changes in the brain elicited by specific stimuli presented to sensory pathways or targeted brain regions. These neurophysiological changes reflect variations in the cognitive functions of the brain. Compared to techniques such as functional magnetic resonance imaging (fMRI) (Rota et al., 2011) and near-infrared spectroscopy (NIRS) (Lim et al., 2020), ERP offers millisecond temporal resolution, enabling precise measurement of cognitive processing. ERP is extracted by repeating the same stimulus across multiple trials and then averaging the EEG recordings. ERP is widely used in psychology, neuroscience, and clinical research to study sensory processes, attention, memory, language, and cognitive functions. Clinically, ERP can be used to diagnose and monitor neurodegenerative diseases, mental disorders, and cognitive impairments (Paitel et al., 2021; Du et al., 2022; MacNamara et al., 2022; Liu et al., 2021; Song et al., 2022).

**Figure 1:**
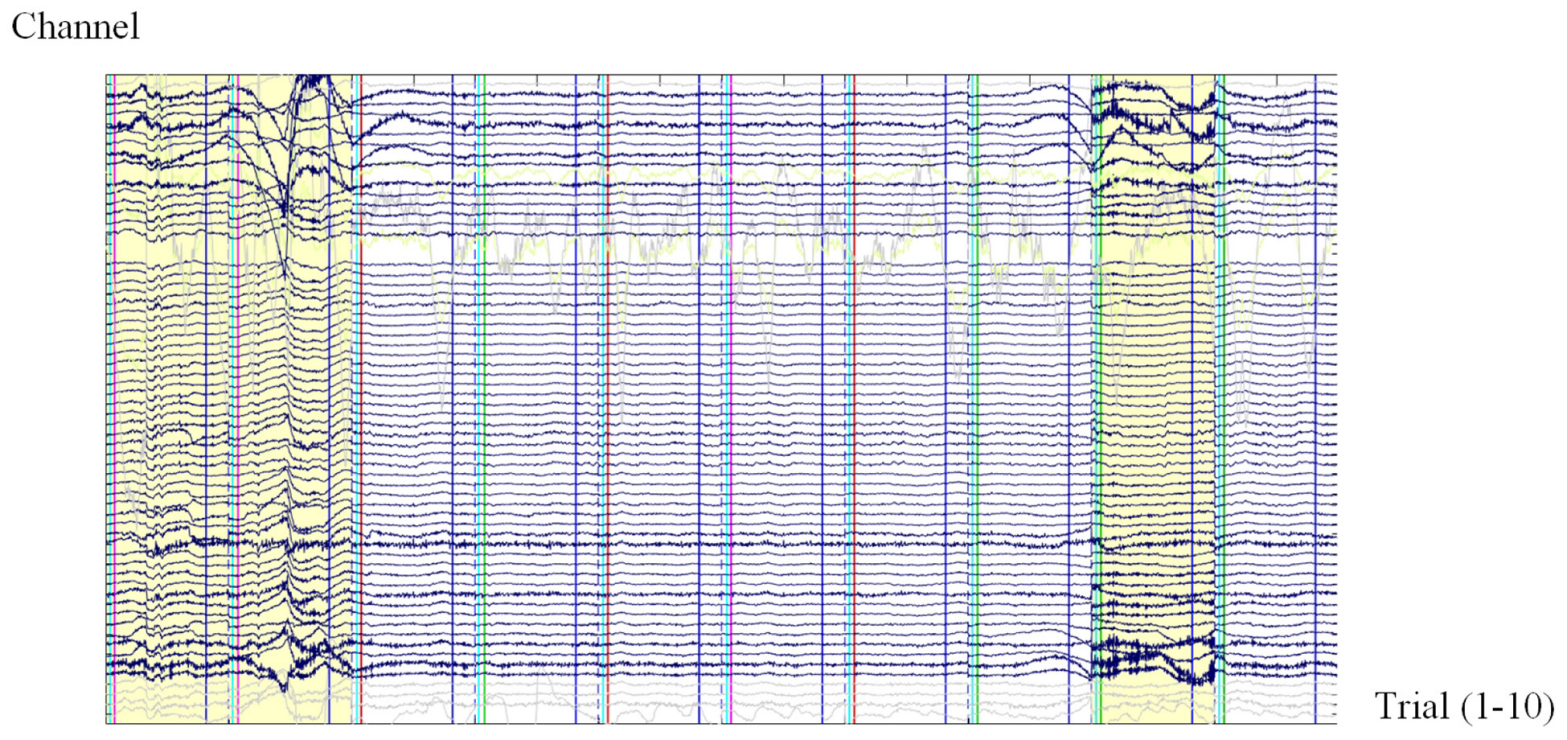
An EEG image containing bad trials displayed using the EPAT toolbox. Each image contains ten independent trials separated by vertical dashed lines (dark blue). The *x*-axis represents trial index (Trials 1–10, from left to right), with each trial containing time-series samples of EEG signals. The *y*-axis represents EEG channels. Gray waveforms indicate previously identified bad channels. The first, second, and ninth trials contain severe artifacts and are marked as bad (highlighted in yellow with red bounding boxes). All subsequent EEG visualization figures in this paper follow the same format and should be interpreted as standardized visual inputs to the YOLOBT model rather than traditional time-series signals.

The quality of data used for processing and analysis is crucial in ERP research. “Bad trials” (Patel et al., 2015; Jas et al., 2017) refer to trials deemed unsuitable for further analysis due to excessive noise or artifacts, as shown in Figure 1. If not identified and handled, bad trials can significantly affect the accuracy and reliability of ERP data analysis, leading to misleading conclusions.

The identification and removal of bad trials presents a critical challenge in ERP preprocessing. Researchers must balance data retention with artifact rejection, often employing adaptive strategies that adjust tolerance thresholds based on overall signal quality. When global data quality is high, stricter criteria are applied; when quality is compromised, more lenient thresholds preserve statistical power while maintaining data integrity. This adaptive approach is especially crucial when confronted with inconsistent data quality, as it helps ensure accurate data analysis and maximizes data utility.

Traditional artifact detection methods (Bhattacharyya et al., 2013; Kauppila et al., 2018; Boudaya et al., 2022; Hermans et al., 2023) operate locally on signal segments, lacking the capability for trial-level global assessment and dynamic threshold adjustment. Recent advances in deep learning (Lawhern et al., 2018; Craik et al., 2019) offer promising solutions for EEG analysis. Deep learning approaches can directly process raw EEG signals (Choo et al., 2023) or transformed representations (Wang et al., 2025; Zhang et al., 2024). Particularly, object detection (Xiao et al., 2020) models applied to visualized EEG waveform images can bypass complex signal processing steps while aligning naturally with expert visual inspection methods (Fraiwan, 2024; Oghabian et al., 2025). You Only Look Once (YOLO) (Xiao et al., 2020), a popular object detection algorithm, shows great potential for this application.

This paper proposes a YOLO-based bad trial detection model, YOLOBT, which focuses on dynamically adjusting classification based on global signal quality. To address the challenge that EEG images significantly differ from common image types as they consist of waveform sequences with complex and subtle features, we improve the YOLOv5 backbone network by designing a Cross-Layer Attention Bottleneck (CLAB), which introduces a cross-layer attention mechanism to enhance the extraction of bad trial features. To more effectively utilize features from different dimensions and accurately locate artifacts of various scales, we propose the Hierarchical Feature Guidance Module (HFGM), which uses high-level features to guide low-level features, better leveraging the information from high-level features. To solve the current algorithm's inability to adjust the artifact detection threshold based on data quality dynamically, we design the Global Information Classification Module (GICM), which enables the network to assess signal quality by reading global information and dynamically adjusting the threshold for bad artifact detection. YOLOBT surpasses the baseline YOLOv5 and other advanced object detection networks, as demonstrated by experiments. The heatmap analysis results suggest that the YOLOBT model employs adaptive assessment strategies that are comparable to those of experts.

## 2 Related work

### 2.1 Bad trial detection

Currently, research on bad trial detection remains relatively limited. Patel et al. (2015) proposed a template-based cross-correlation method that selects high-correlation trials by calculating the correlation coefficient between individual trials and an average template. However, this method relies on static thresholds and is highly sensitive to contamination of the initial template, resulting in insufficient adaptability to handle fluctuations in data quality. Jas et al. (2017) further advanced this field by introducing automated threshold selection and sensor-specific adjustment strategies. Nevertheless, this approach overly focuses on local noise detection at the sensor level, failing to adequately consider the overall relationships at the trial level. For the specific task of bad trial selection, both methods mentioned above attempt to introduce global considerations, but they remain limited in effectiveness due to the constraints of traditional signal processing techniques. With the rapid development of deep learning technology, its outstanding capabilities in image and signal processing domains show promise in providing more suitable methods and global strategies for bad trial detection tasks.

### 2.2 EEG waveform image analysis

EEG data analysis commonly employs complex signal transformation methods, while in recent years, researchers have begun to explore the direct use of EEG waveform images. This approach aligns with the visual inspection process of experts, effectively bypassing complex signal processing and feature engineering steps, thereby increasing the generalizability of models. Fussner et al. (2024) utilized CNN combined with transfer learning to analyze raw EEG images, successfully distinguishing between epileptic and non-epileptic seizures, achieving accuracy rates of 86.9 and 87.3% on datasets from two independent medical institutions. Emami et al. (2019) demonstrated that CNN can detect epileptic seizures through EEG image analysis, with a detection rate of 100% per minute, significantly outperforming commercial software such as BESA (73.3%) and Persyst (81.7%). Muñoz et al. (2022) proposed a computational model based on raw EEG images and transfer learning for detecting epileptiform events in pediatric EEG, achieving accuracy rates exceeding 95% in binary classification tasks and over 87% in multi-classification tasks. Tosun and Kasım (2020) pioneered the application of semantic segmentation algorithms to EEG blink artifact detection, converting multi-channel EEG recordings into images for analysis, achieving a classification accuracy of 94.4%. Furthermore, heatmap results indicated that the model's classification approach highly corresponds with the visual inspection methods of experts.

### 2.3 Object detection in EEG waveform image analysis

In recent years, the rapid development of deep learning technology has driven significant progress in the field of object detection. Object detection has achieved breakthroughs in domains such as agricultural monitoring (Kamalesh Kanna et al., 2024), medical image analysis (Mushtaq et al., 2022; Santos et al., 2022), and industrial automation (Yang et al., 2023), matching or surpassing human-level performance in many tasks. Object detection has also been successfully applied to EEG waveform image analysis, significantly enhancing the efficiency and accuracy of EEG feature recognition.

In epilepsy detection, Oghabian et al. (2025) proposed a method based on YOLOv3, v4, and v7 for detecting multiple types of epileptic waveforms. Among four classification strategies, YOLOv4 demonstrated the best performance, with average sensitivity, specificity, and accuracy reaching 96.7%, 94.3%, and 92.8%, respectively. In the field of sleep-related EEG waveform detection, Khasawneh et al. (2022) developed a high-precision K-complex detection system based on Faster R-CNN and various CNN feature extraction backbones. VGG19 and Inceptionv3 models performed best in different testing scenarios, achieving accuracy up to 99.8%. Similarly, Fraiwan (2024) employed visual detection methods to simultaneously identify K-complexes and sleep spindles in EEG, comparing Faster R-CNN, YOLOv4, and YOLOX. Results indicated that this approach could achieve average precision exceeding 95% under standard thresholds.

These studies demonstrate that applying object detection techniques to EEG waveform image analysis can significantly improve detection accuracy and simulate expert visual inspection methods. In particular, the YOLO series algorithms, with their efficiency and precision, show tremendous potential in EEG image analysis. Despite YOLO's excellent performance in object detection, its architecture is primarily based on CNN, which has inherent limitations. CNN's limited receptive field restricts its ability to capture long-range spatial dependencies (Xiao et al., 2020), while bad trial determination requires comprehensive consideration based on global image quality. Such global information is crucial for simulating experts' dynamic adjustments of bad trial determination criteria.

### 2.4 Self-attention mechanism

The self-attention mechanism provides a new approach to address this issue. The Transformer model (Vaswani et al., 2017), based on self-attention, effectively captures global relationships between features, enabling excellent performance in tasks requiring long-range dependencies. Self-attention overcomes CNN's limitations in acquiring global information by calculating the associations between all positions in a sequence, establishing direct connections between elements.

Recent studies have demonstrated the advantages of the global properties of self-attention mechanisms across various computer vision tasks. Research Huang et al. (2023) in vehicle re-identification leveraged the self-attention mechanism to focus on dependencies between different vehicle parts, capturing both global and local features through a coarse-to-fine attention decomposition, thus reducing reliance on expensive local feature extraction. Study (Liang et al., 2023) in remote sensing building change detection fused dynamic and static contexts, enabling the model to break the limitation of the receptive field and perceive image change features globally. Its dual attention mechanism processed high-frequency and low-frequency information separately, comprehensively capturing change patterns at different scales. Li et al. (2023) in video inpainting utilized the global modeling capability of self-attention to calculate more accurate spatial-temporal dependencies, maintaining global context awareness while improving computational efficiency through a local-global interleaving design. In medical image analysis, the MEDUSA architecture proposed in study (Aboutalebi et al., 2022) enabled the network to establish global connections across multiple levels of abstraction through its “single body, multi-scale heads” implementation of self-attention, effectively capturing subtle disease characteristics and overlapping appearances between different diseases.

These studies indicate that the global modeling capability of self-attention mechanisms can help systems overcome the limitations of traditional CNN architectures, providing effective solutions for tasks requiring comprehensive global information for decision-making, with significant implications for EEG bad trial detection tasks.

## 3 Method

Before introducing the model architecture, it is important to clarify YOLOBT's position within the EEG preprocessing pipeline. Following the workflow established in the EPAT toolbox (Shi et al., 2024), bad trial detection occurs as the final quality control step, after filtering and ICA artifact correction, and before ERP averaging. Trials reaching the bad trial detection stage have already undergone ICA correction. Those identified as “bad” by YOLOBT contain severe, unrecoverable artifacts that cannot be corrected through filtering or ICA. These trials must be excluded to ensure ERP quality. Therefore, YOLOBT serves as a final quality assurance step that complements rather than replaces conventional artifact correction methods.

**Figure 2:**
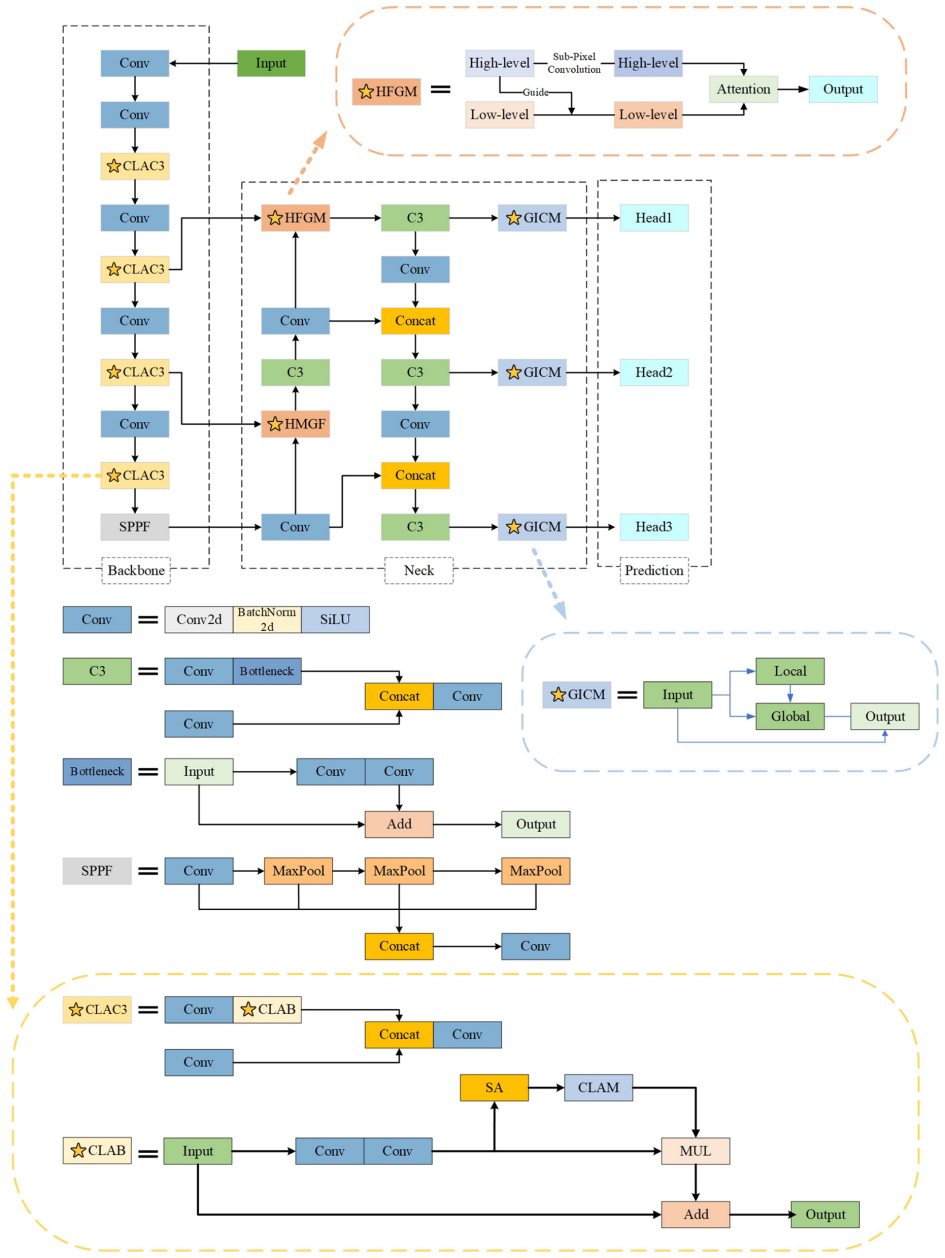
The pipeline of the proposed YOLOBT. Different colors represent distinct functional modules in the figure. We have improved the original YOLOv5 by incorporating CLAB (CLAC3), HFGM, and GICM, marked with pentagrams.

This section introduces the design of the YOLOBT model. We select YOLOv5 as the base model and introduce three key modifications to make it more suitable for detecting bad trials. The overall architecture of YOLOBT is shown in Figure 2.

First, for the C3 module, we not only improve it but also add attention mechanisms to each of its levels. With this design, the model can more effectively focus on processing critical information. Additionally, we introduce an innovative attention fusion module that optimizes information flow, ensuring the model fully utilizes information from previous layers. This improvement promotes deep integration of features, thereby enhancing the model's ability to capture details, which is crucial for artifact feature extraction.

Furthermore, we design the HFGM to efficiently utilize and smoothly integrate features by using high-level features to guide low-level features. This dynamic fusion process ensures that high-level features are fully utilized while strengthening the synergy between different levels of features. In this way, the model can form richer and more multidimensional feature representations, greatly enhancing its ability to recognize artifacts of varying sizes in complex scenarios.

Finally, addressing the specificity of the bad trial detection task, we design the GICM, positioning it before the prediction head. This allows the model to dynamically adjust the artifact detection threshold based on varying signal quality. The module effectively interacts between local and global features by using convolutional layers to guide the weight distribution of self-attention in the Transformer. This combines the local feature extraction capabilities of CNN with the global feature extraction advantages of Transformers. Through this module, the model can simulate the strategy of manually selecting bad trials, improving its practicality.

### 3.1 Cross-layer attention bottleneck

EEG images differ significantly from common image types, primarily due to their content, which usually consists of complex waveform sequences. These waveforms are not only intricate but also subtle, making it challenging for conventional image-processing models to effectively recognize and handle them. Moreover, EEG image data often contain numerous small perturbations that can affect the accuracy and stability of models in extracting key artifact features. Therefore, targeted improvements to the traditional YOLOv5 model are crucial for effectively extracting artifact features. Artifacts in EEG images can originate from various factors, such as physiological responses, technical issues, and environmental interference, resulting in significant intra-class variability. This necessitates a more robust backbone network to extract features from EEG images for better artifact recognition.

**Figure 3:**
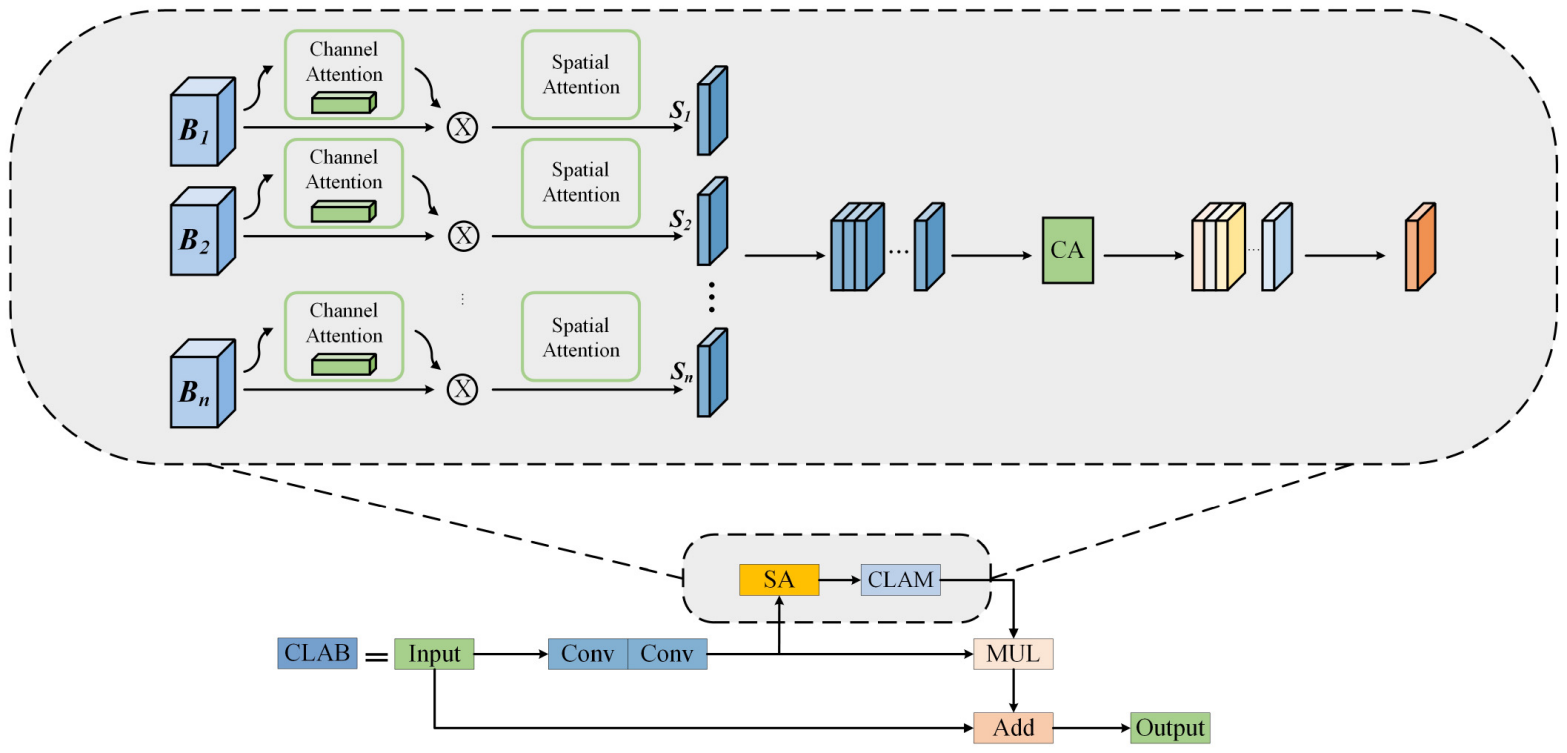
The structure of CLAB. *S*_1_...*S*_*n*_ represent the spatial attention maps generated by the nth bottleneck layer. These attention maps from different layers are concatenated and their weights are redistributed using a channel attention module. Finally, a 1x1 convolution is applied to fuse them into a single-channel attention map.

When manually selecting bad trials, researchers focus on regions containing artifacts. Based on this mechanism, we introduce a spatial attention mechanism into the backbone network to significantly enhance the network's ability to perceive and extract important artifact features, thereby improving the feature extraction capability of the backbone network. Additionally, we design a cross-layer attention mechanism to integrate spatial attention maps from different layers, further strengthening the feature extraction capability of the backbone network. The structure of the CLAB is shown in Figure 3.

Step 1: Firstly, by introducing a spatial attention mechanism, we enable the model to focus on the most critical areas in the EEG signal map, generating spatial feature maps in preparation for subsequent processing.

Step 2: Based on the extracted spatial attention feature maps, we further fuse these feature maps with spatial attention feature maps obtained from all previous layers. Since attention information from different layers may contain redundancy and varying importance levels, simple stacking or addition may not achieve optimal results. Therefore, we introduce a new continuous cross-layer attention transfer mechanism. This mechanism effectively integrates spatial attention maps from different bottleneck layers and assigns corresponding weights based on the importance of each layer. Using this method, we ultimately generate a new, information-rich spatial feature map.

Step 3: The new spatial feature map, which includes all the previously obtained information is multiplied by the original input.

In the following sections, we will introduce the spatial attention (SA) and the Cross-Layer Attention Module (CLAM) separately.

#### 3.1.1 Spatial attention

Traditional object detection methods are relatively weak at filtering key features, which may lead to model overfitting and limited generalization ability (Li et al., 2021). In recent years, attention mechanisms have been added to convolutional neural networks, improving the performance of object recognition. The attention mechanism plays a crucial role in modern neural network design by mimicking human cognitive awareness of specific information to enhance model performance and efficiency. Attention mechanisms enable neural networks to automatically distinguish between key features and those that are secondary or irrelevant. In this way, the attention mechanism helps the network focus on the most critical information for completing specific tasks, ignoring distracting information that does not positively impact the outcome. For EEG images, the spatial attention mechanism allows the model to focus on the most important areas of the EEG signal map, emphasizing the extraction of waveforms at these locations and enhancing the extraction and capture of artifact features.

In this paper, we employ the Convolutional Block Attention Module (CBAM) (Woo et al., 2018) and integrate it into the bottleneck of each layer to enhance the feature extraction capability of each layer. CBAM, by concatenating channel and spatial attention modules, enables the model to better focus on more critical features. The channel attention module aims to emphasize the feature channels that are more important for the current task and reduce the influence of unimportant channels. For a given CLAC3 module, we define the feature map after the *n*-th layer CLAB convolution as $B_{n}\in {\mathbb{R}}^{C\times H\times W}$. By utilizing global average pooling and global max pooling, the feature map of size *C*×*H*×*W* is converted into two one-dimensional vectors of size *C*×1 × 1. These vectors are fed into a shared two-layer neural network, specifically a multilayer perceptron (MLP). The shared network learns the importance weights of each channel, which are then activated by a sigmoid function to obtain the one-dimensional channel attention map *M*_*c*_*n*_. Finally, these weights are used to adjust the channels of the original feature map.

The calculation formula is shown below, where σ represents the sigmoid function. Avg(·) represents global average pooling, and Max(·) represents global max pooling.


$$\begin{array}{l} M_{c\text{\_}n}=\sigma \left(\text{MLP}\left(\text{Avg}\left(B_{n}\right)\right)+\text{MLP}\left(\text{Max}\left(B_{n}\right)\right)\right) \end{array}$$ (1)

$$\begin{array}{l} B_{n}^{\prime }=M_{c\text{\_}n}⊗B_{n} \end{array}$$ (2)

After channel attention adjustment, to further focus on important areas at different spatial positions in the feature map and enhance the model's ability to recognize key parts of the image, we perform average pooling and max pooling operations on the adjusted feature map along the channel dimension. The results of the pooling operations are stacked along the channel dimension. Then, these stacked data are processed by a 7 × 7 convolution kernel, converting them into a single-channel feature map. Subsequently, this feature map is processed by a sigmoid function, generating the final spatial attention feature map *S*_*n*_.

The calculation formula is shown below, where *f*^7 × 7^ represents a convolution kernel with a size of 7 × 7.


$$\begin{array}{l} S_{n}=\sigma \left(f^{7\times 7}\left(\left[\text{Avg}\left(B_{n}^{\prime }\right);\text{Max}\left(B_{n}^{\prime }\right)\right]\right)\right) \end{array}$$ (3)

#### 3.1.2 Cross-layer attention module

Through the above operations, we obtain the spatial attention map *S*_*n*_ of the *n*-th layer CLAB. To integrate the attention information from the previous *n*−1 layers, we introduce the CLAM.

For the CLAB module of the *n*-th layer bottleneck, we concatenate the spatial attention maps {*S*_1_, *S*_2_, ..., *S*_*n*−1_} generated from the previous *n*−1 layers with the spatial attention map *S*_*n*_ produced by the current layer. Although spatial attention maps from different layers carry a wealth of information, they still contain some redundant or misleading information. To more efficiently extract and utilize the attention information from these layers, we introduce the Channel Attention (CA) module (Hu et al., 2018). This module assigns different weights to the feature maps from various layers, selecting and enhancing the more valuable attention maps to improve the effectiveness and accuracy of the information, thereby optimizing its utilization. For the weighted attention maps, we use a 1 × 1 convolution to fuse them into a single-channel attention map.

The Channel Attention module enhances the network's ability to model inter-channel dependencies by introducing the “Squeeze-and-Excitation” (SE) structure (Hu et al., 2018). This structure primarily improves the network's expressive power by recalibrating the feature responses of the channels. The workflow of the SE module can be divided into two main steps.

For a feature map *U* with dimensions *H*×*W*×*C*, global average pooling is performed on each channel to generate channel descriptors. The global average pooling output *z*_*c*_ for each channel *c* is calculated as follows:


$$\begin{array}{l} z_{c}=\frac{1}{H\times W}\overset{W}{\underset{i=1}{\sum }}\overset{H}{\underset{j=1}{\sum }}u_{c}\left(i,j\right) \end{array}$$ (4)

where *u*_*c*_(*i, j*) represents the feature value at position (*i, j*) in the *c*-th channel.

Next, *z* undergoes the excitation step. The excitation step uses an MLP to learn the non-linear interactions that capture inter-channel dependencies from global information.


$$\begin{array}{l} s_{c}=\sigma \left(W_{2}\text{ReLU}\left(W_{1}z\right)\right) \end{array}$$ (5)

where *W*_1_ and *W*_2_ are the weight matrices of the MLP, and σ represents the sigmoid function. Finally, the weights generated by the excitation operation are applied to the corresponding channels in the original feature map *U* to achieve feature re-weighting.


$$\begin{array}{l} {ũ}_{c}=s_{c}\cdot U_{c} \end{array}$$ (6)

The specific formula for the CLAB module is as follows, where *Final*_*S*_*n* represents the final generated weighted fused single-channel spatial attention map, SE(·) represents the SE attention mechanism, and Cat(·) represents the concatenation operation.


$$\begin{array}{l} Final\text{\_}S\text{\_}n=g_{1\times 1}\left(\text{SE}\left(\text{Cat}\left(S_{1},S_{2},\cdots \;,S_{n}\right)\right)\right) \end{array}$$ (7)

Finally, we perform element-wise multiplication between the obtained *Final*_*S*_*n* and the *n*-th layer's *B*_*n*_ to obtain the output. To make the output smoother, we then apply a Channel Attention (CA) module to enhance the quality of the output features.


$$\begin{array}{l} B_{n\text{\_}output}=\text{SE}\left(Final\text{\_}S\text{\_}n⊗B_{n}\right) \end{array}$$ (8)

### 3.2 Hierarchical feature guidance module

YOLOv5 adopts the Feature Pyramid Network (FPN) architecture to accurately handle multi-scale information, aiming to enhance the detection accuracy of small objects while maintaining the ability to recognize large objects. However, this fusion strategy has two drawbacks.

**Figure 4:**
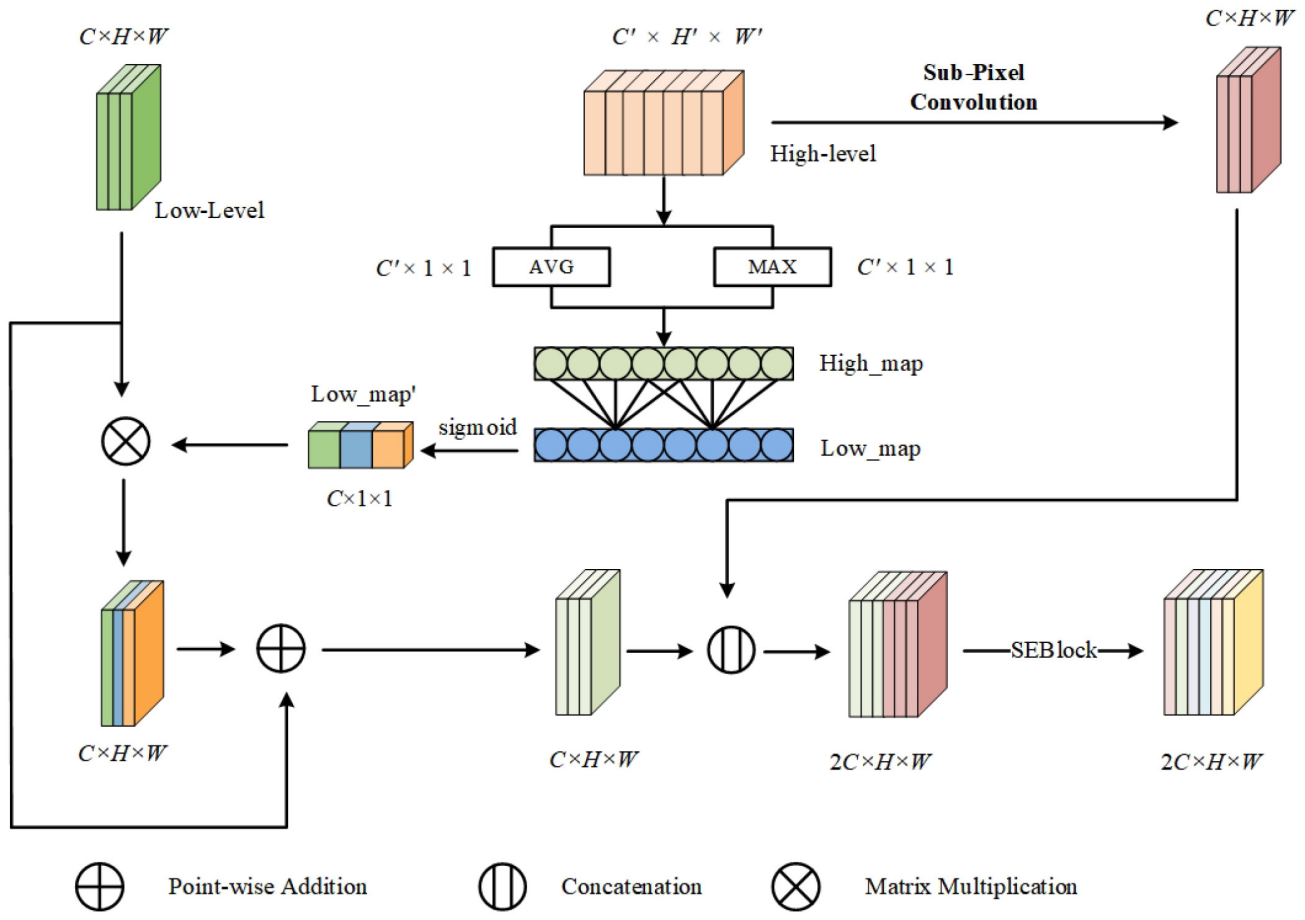
The structure of HFGM. High-level features first guide low-level features, and then dynamic upsampling is performed using sub-pixel convolution. Finally, the two are concatenated and fused through an SEBlock.

Firstly, YOLOv5 does not fully utilize high-level feature maps. High-level feature maps contain rich feature information that can be used to guide lower-level feature maps, thereby enabling more precise localization of artifacts. Secondly, YOLOv5 employs interpolation upsampling for high-level feature maps. While this method is simple and efficient, it mainly operates based on local pixel values and does not fully exploit and utilize the global contextual information in the image to optimize the upsampling results. For complex and highly variable data like EEG images, this non-learning-based upsampling method is insufficient to maintain image quality and provide optimal upsampling effects. Therefore, a learning-based upsampling method that better understands image content and structure is needed. To address the above two shortcomings, we propose the HFGM. The network structure of this module is shown in Figure 4.

To effectively guide low-level features with high-level features, we perform global average pooling and global max pooling on the high-level features separately. The resulting features are then flattened and concatenated to obtain *Y*.


$$\begin{array}{l} High\text{\_}map=\text{concat}\left(Output_{MAX},Output_{AVG}\right) \end{array}$$ (9)

*High*_*map* contains rich semantic information from the high-level features. We map *High*_*map* so that its dimension is equal to the number of channels in the low-level feature map, obtaining *Low*_*map*. Finally, we use the sigmoid function to map *Low*_*map* to a range between 0 and 1, resulting in *Low*_*map*′.


$$\begin{array}{l} Low\text{\_}map^{\prime }=\text{sigmoid}\left(\text{conv}\left(Low\text{\_}map\right)\right) \end{array}$$ (10)

After obtaining *Low*_*map*′, we multiply it with the low-level features, using the high-level features to guide the low-level features.


$$\begin{array}{l} Low\text{\_}level^{\prime }=\left(Low\text{\_}level⊗Low\text{\_}map^{\prime }\right)⊕Low\text{\_}level \end{array}$$ (11)

As previously mentioned, the interpolation upsampling method, although simple and efficient, primarily operates based on local pixel values and may not fully exploit and utilize the global contextual information in the image to optimize the upsampling results. Therefore, we use sub-pixel convolution (Shi et al., 2016) instead of interpolation upsampling. Sub-pixel Convolution is a technique used to increase image resolution and is commonly applied to super-resolution reconstruction tasks in deep learning. It allows the model to generate high-resolution output from lower-resolution images by performing a rearrangement operation rather than direct convolution to achieve resolution enhancement. Specifically, for the high-level features *High*_*level*∈*H*′ × *W*′ × *C*′, we first map the number of channels to *C*×*r*^2^, resulting in *High*_*level*′∈*H*′ × *W*′ × *Cr*^2^, where *C* is the number of channels in the low-level features (*Low*_*level*) and *r* is the upsampling factor (the resolution enhancement factor, *r* = *H*/*H*′). The sub-pixel convolution layer transforms *High*_*level*′ into an output image of size *rH*′ × *rW*′ × *C*. Each *r*^2^ feature block is mapped to a single pixel in the output image, and these *r* channels are distributed across the spatial dimensions of the output image. This process can be regarded as a depth-to-space transformation.

After performing the upsampling operation using sub-pixel convolution, higher-resolution high-level features with better image quality are obtained.

Next, the upsampled high-level features are concatenated with the low-level features that have been optimized under the guidance of the high-level features. Then, an SENet (Hu et al., 2018) is applied to reweight the concatenated features, dynamically adjusting the relative importance of the feature channels to enhance the fusion of low-level and high-level features.


$$\begin{array}{l} Output=\text{SENet}\left(\text{cat}\left(Low\text{\_}level^{\prime },High\text{\_}level_{upsample}\right)\right) \end{array}$$ (12)

where *Low*_*level*′ denotes the low-level features guided by the high-level features, *High*_*level*_*upsample*_ denotes the high-level features after sub-pixel convolution, cat(·) denotes the concatenation operation, and SENet(·) denotes the SENet module.

### 3.3 Global information classification module

As mentioned earlier, in ERP research, researchers adjust the criteria for evaluating local artifacts based on the global condition of the global data quality. This approach reflects the brain's advantage in global analysis when processing complex information. Specifically, the brain, when faced with a large amount of information, can flexibly adjust its attention to details based on the global context, which is crucial when handling artifact-contaminated ERP data. By globally assessing data quality and adjusting the tolerance for artifacts in individual trials accordingly, researchers can achieve an optimal balance between ensuring data quality and maximizing data utilization.

**Figure 5:**
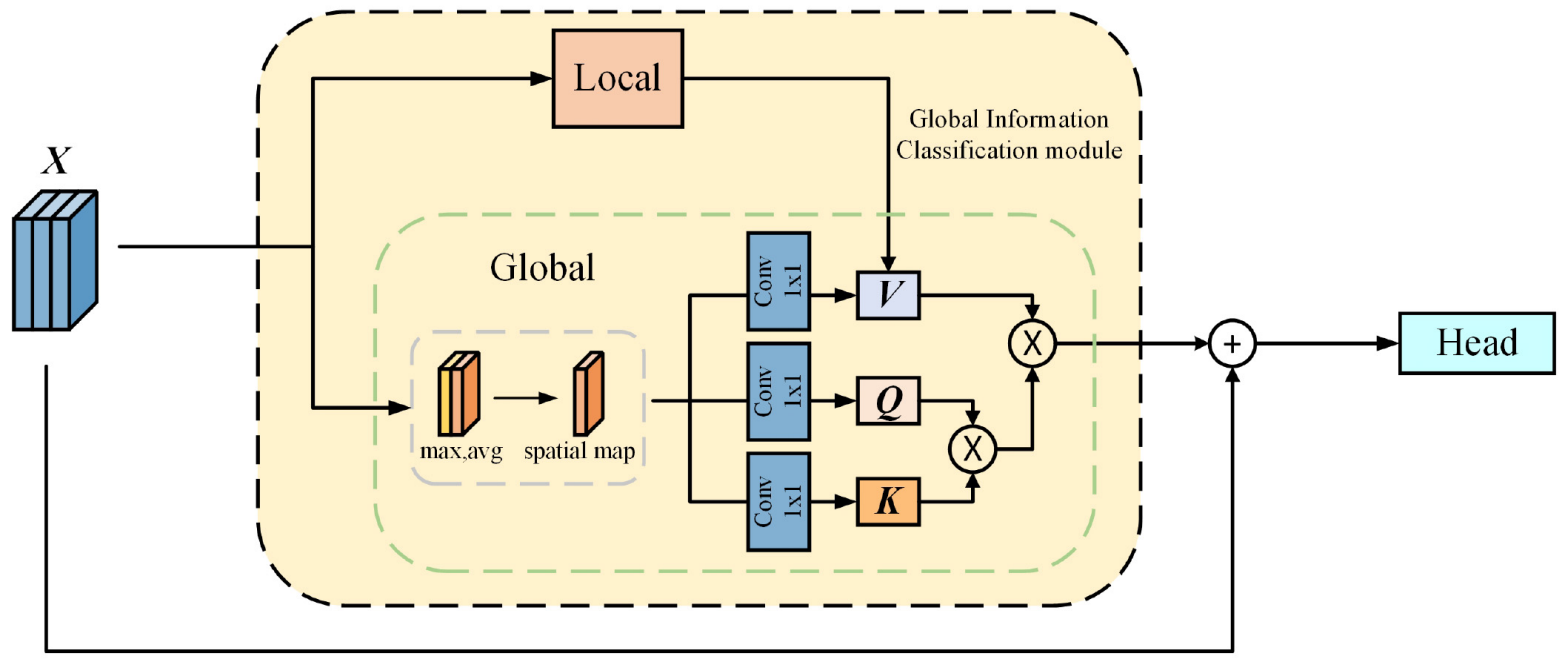
The structure of GICM, consisting of global and local branches. It integrates local information into global information to better evaluate EEG signal quality, enabling dynamic adjustment of classification results.

Drawing on the methods researchers use to handle bad trials, we have innovatively improved the YOLOv5 deep learning model by introducing a GICM. This module aims to impart more brain-like characteristics to artificial intelligence by emulating the human brain's ability to process global and local information. Through this improvement, the model can consider the details of local features during the recognition process while optimizing the interpretation and judgment of these local features based on global information. The network architecture of the GICM is shown in Figure 5. In this module, we first divide the input tensor into two branches: the global branch and the local branch, aiming to integrate local information into global information to better evaluate the quality of EEG signals.

#### 3.3.1 Local branch

**Figure 6:**
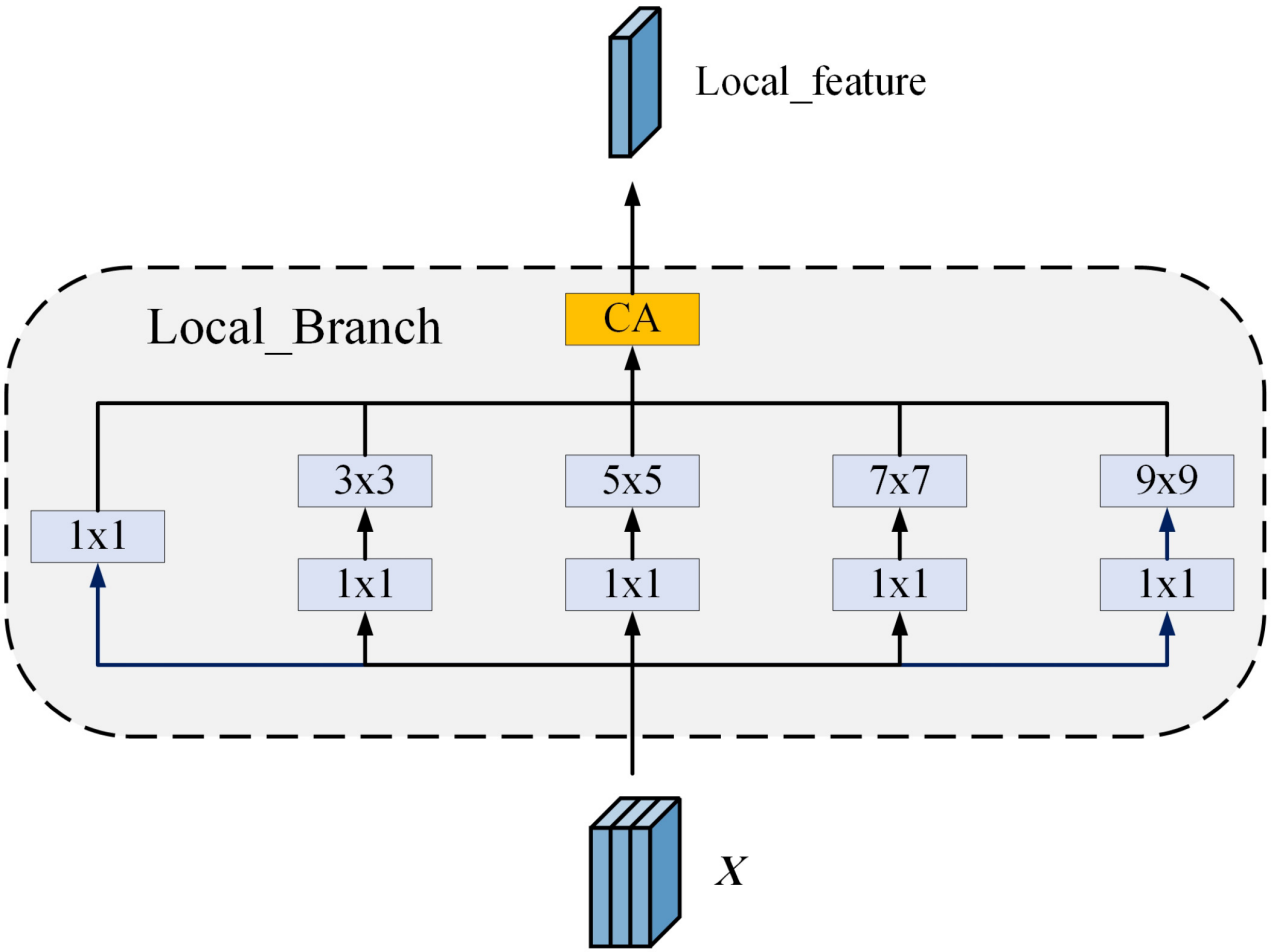
The structure of the local branch. The branch includes five sub-branches, each using convolutional kernels of different sizes to extract local features at multiple scales. A channel attention module is employed to reduce information redundancy.

In our design of the local branch module, we adopt the multi-scale convolutional approach of the Inception network (Szegedy et al., 2015) to effectively capture artifact features of different scales in the image, as shown in Figure 6. This design allows the local branch to provide detailed and discriminative features, crucial for capturing small or differently scaled artifacts in the image. Specifically, this module contains five branches, each using convolutional kernels of different sizes (1 × 1, 3 × 3, 5 × 5, 7 × 7, and 9 × 9), thus extracting rich local features at various scales.

Since each branch independently extracts features, there may be overlapping or redundant information among them. To address this issue and optimize feature representation, we add a channel attention module after merging the feature maps from all branches.

To further prepare for integration with the global feature branch, we use a 1 × 1 convolutional kernel to map the multi-channel data to a single channel. Finally, through a sigmoid activation function, we normalize the output of the 1 × 1 convolution to a range of 0 to 1, obtaining the final local feature map.


$$\begin{array}{l} L\text{\_}feature=\text{Local}\left(X\right) \end{array}$$ (13)

#### 3.3.2 Global branch

Since the core purpose of this module is to adjust the threshold for artifact detection and focus on areas where artifacts may appear, we utilize spatial attention on the feature map before inputting it into the global branch. This allows us to concentrate on specific regions that may be identified as artifacts. Consequently, the subsequent model can adjust the artifact detection threshold based on a comprehensive evaluation of the global signal quality, enabling more precise artifact reassessment.


$$\begin{array}{l} X\text{\_}global=\text{Spatial\_attention}\left(X\right) \end{array}$$ (14)

Next, to more comprehensively capture and analyze the quality of the input EEG signals, we introduce a self-attention mechanism. With its excellent global perception capabilities, the self-attention mechanism can extensively capture contextual information and conduct a thorough evaluation of the entire input signal's quality. Consequently, the model can dynamically and flexibly adjust the artifact detection threshold based on the global characteristics of the signal, thereby simulating the manual method of removing bad trials. When processing the feature map, we use a 1 × 1 convolutional kernel, effectively transforming the feature map into the three components required by the self-attention mechanism: Key, Query, and Value. The Value component carries the primary information of the EEG signal, becoming crucial for subsequent processing.

To enrich the information contained in the Value component, we interact with the local branch. This interaction involves multiplying the feature map extracted by the local branch with the Value component, allowing the information obtained by the local branch to supplement the Value, achieving an effective integration of global and local information. The specific formula is as follows, where Inception(·) denotes the Inception module, Conv(1 × 1) denotes the 1 × 1 convolution kernel, and $\sqrt{d_{k}}$ is the dimension of *K*.


$$\begin{array}{l} K={\text{Conv}}_{1\times 1}\left(X_{global}\right) \end{array}$$ (15)

$$\begin{array}{l} Q={\text{Conv}}_{1\times 1}\left(X_{global}\right) \end{array}$$ (16)

$$\begin{array}{l} V=L\text{\_}feature⊗{\text{Conv}}_{1\times 1}\left(X_{global}\right) \end{array}$$ (17)

$$\begin{array}{l} X_{output}=\text{softmax}\left(\frac{Q\times K^{T}}{\sqrt{d_{k}}}\right)\times V \end{array}$$ (18)

## 4 Results

### 4.1 Dataset

This study utilized a private dataset for training. EEG data were collected using a 64-channel EGI system, employing a dot probe paradigm in which subjects were required to rapidly detect and locate probe dots appearing at previous stimulus positions to evaluate attentional bias and cognitive processing. The dataset includes recordings from 241 subjects (231 patients with Alzheimer's disease and 10 patients with epilepsy), with each subject completing approximately 240 trials. Both patient groups undergo ERP-based cognitive assessment as part of clinical evaluation at our collaborating hospitals, and their data were combined to increase sample size and data diversity. These clinical recordings inherently contain diverse naturally occurring artifacts representative of real-world EEG acquisition scenarios, including signal noise, eye movement artifacts, electromyographic (EMG) contamination, and movement-related artifacts. Unlike artificially controlled conditions, this clinical dataset reflects the true complexity encountered in practical EEG analysis. Bad trials were annotated by an experienced neurophysiologist through visual inspection. We used the EPAT toolbox (Shi et al., 2024) to visualize the EEG data, displaying ten trials per screen, and each screen served as one input image for the model. Prior to bad trial annotation, bad channels were identified and marked, with their waveforms displayed in gray. Ultimately, we constructed a dataset containing over 2,600 EEG images and conducted model training and testing on this dataset.

### 4.2 Implementation details

This study was conducted on an Ubuntu 20.04 64-bit system, which was equipped with 120 GB of RAM, powered by 12 vCPU Intel(R) Xeon(R) Platinum 8352V CPUs operating at 2.10GHz, and accelerated by an RTX 4090 GPU featuring 24GB of VRAM. For the deep learning tasks, we adopted the PyTorch framework. The detailed training hyperparameters are presented in Table 1, following the standard YOLOv5 configuration which has been extensively validated across object detection tasks. The reported performance metrics were obtained on the held-out test set using the model checkpoint with the best validation mAP. For ablation and comparison experiments, each model was trained independently from scratch to ensure fair comparison.

**Table 1:** Training hyperparameters.

**Hyperparameter**	**Value**
Epochs	600 (early stopping patience = 50)
Batch size	16
Initial learning rate	0.01
Optimizer	SGD (momentum = 0.937)
Weight decay	0.0005
LR scheduler	Cosine annealing
Input resolution	640 × 640 pixels
Data augmentation	Mosaic, mixup (α = 0.2)
Train/val/test split	70%/15%/15%

### 4.3 Metrics

In object detection, the performance of a model is typically evaluated using Precision and Recall:


$$\begin{array}{l} Precision=\frac{TP}{TP+FP} \end{array}$$ (19)

$$\begin{array}{l} Recall=\frac{TP}{TP+FN} \end{array}$$ (20)

where TP, FP, and FN represent the number of true positive, false positive, and false negative samples, respectively.

Average precision (AP) is the area under the Precision-Recall curve for a particular class, with a higher AP indicating better model performance. The AP is calculated as:


$$\begin{array}{l} AP=\underset{n}{\sum }\left(R\left(n\right)-R\left(n-1\right)\right)P\left(n\right) \end{array}$$ (21)

where *R*(*n*) is the Recall at the *n*-th threshold, *R*(*n*−1) is the Recall at the *n*−1-th threshold, *P*(*n*) is the Precision at the *n*-th threshold.

Mean Average Precision (mAP) is the average of the areas under the P-R curves for all classes. It is obtained by averaging the AP of each class. The mAP Score is calculated as:


$$\begin{array}{l} mAP=\frac{1}{C}\overset{C}{\underset{c=1}{\sum }}AP\left(c\right) \end{array}$$ (22)

where C is the number of target classes.

F1 Score, which concurrently considers both the Precision and Recall of a model, represents the harmonic mean of these two metrics. A higher F1 Score indicates superior model performance. The F1 Score is calculated as:


$$\begin{array}{l} F1score=2\times \frac{Precision\times Recall}{Precision+Recall} \end{array}$$ (23)

In object detection, Frames Per Second(FPS) serves as a crucial metric for evaluating the performance of an algorithm or model. Specifically, it denotes the number of image frames the object detection algorithm or model can process per second. This metric directly reflects the speed and efficiency of the algorithm.

### 4.4 Ablation experiments

**Figure 7:**
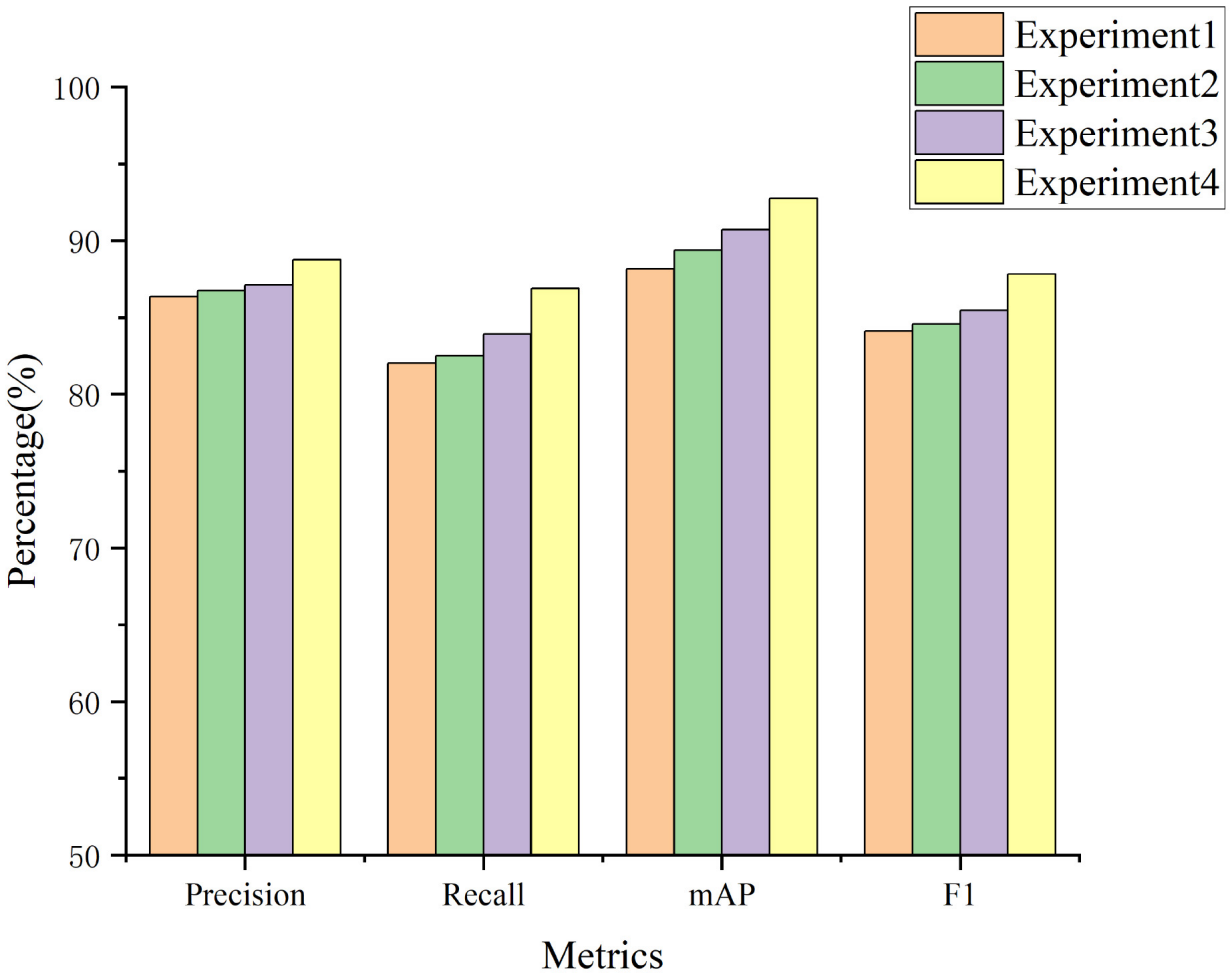
Precision, recall, mAP, and F1 scores from the ablation experiments.

To evaluate the model's effectiveness, ablation experiments were designed to demonstrate the effectiveness of each module in YOLOBT. The design of the ablation experiments is shown in Table 2, and the results are shown in Table 3 and visualized in Figure 7 through bar charts for clearer comparison.

**Table 2:** Ablation experiment idea on our dataset.

**No**	**YOLOv5s**	**CLAB**	**HFGM**	**GICM**
Experiment 1	✓			
Experiment 2	✓	✓		
Experiment 3	✓	✓	✓	
Experiment 4	✓	✓	✓	✓

**Table 3:** The results of ablation experiments on our dataset.

**No**	**Pre (%)**	**Rec (%)**	**mAP (%)**	**F1 (%)**
Experiment 1	86.36	82.01	88.16	84.13
Experiment 2	86.76	82.50	89.39	84.58
Experiment 3	87.12	83.92	90.73	85.49
Experiment 4	**88.76**	**86.89**	**92.76**	**87.82**

Bold values indicate the best performance for each metric.

The experiments are designed as follows: Experiment 1 uses the basic YOLOV5s model to process EEG images, serving as the baseline model. Experiment 2 replaces the traditional YOLOv5 backbone with the CLAB. Experiment 3 uses the CLAB and HFGM. Experiment 4 uses the CLAB, HFGM, and GICM.

In Experiment 2, the model introduced CLAB, resulting in improvements in four evaluation metrics—Pre, Rec, mAP, and F1 score—by 0.30%, 0.49%, 1.23%, and 0.45%, respectively, compared to the baseline YOLOv5 in Experiment 1. CLAB enhances the transmission of information across different layers through cross-layer attention, thereby strengthening the feature extraction capability of the backbone network and improving detection performance. In Experiment 3, with the further addition of HFGM, the four evaluation metrics increased by 0.36%, 1.42%, 1.34%, and 0.91%, respectively, compared to Experiment 2. This demonstrates that HFGM effectively utilizes the features extracted by CLAB, using high-level features to guide low-level features, thereby improving the results. In Experiment 4, compared to the baseline YOLOv5, the four metrics increased by 2.40%, 4.88%, 4.60%, and 3.69%, respectively. Compared to Experiment 3, the metrics increased by 1.64%, 2.97%, 2.03%, and 2.33%, respectively. GICM dynamically adjusts the threshold for identifying bad trials based on the quality of EEG signals, lowering the tolerance for artifacts in high-quality EEG images and increasing the tolerance in low-quality EEG images, significantly enhancing Pre and Rec, and further optimizing the model's performance in detecting bad trials in EEG. These results demonstrate the effectiveness of the YOLOBT model in detecting bad trials in EEG through the use of CLAB, HFGM, and GICM.

### 4.5 Comparison experiments

In this section, we compared YOLOBT with three categories of methods: classical object detection models, traditional signal processing methods, and temporal deep learning methods.

**Figure 8:**
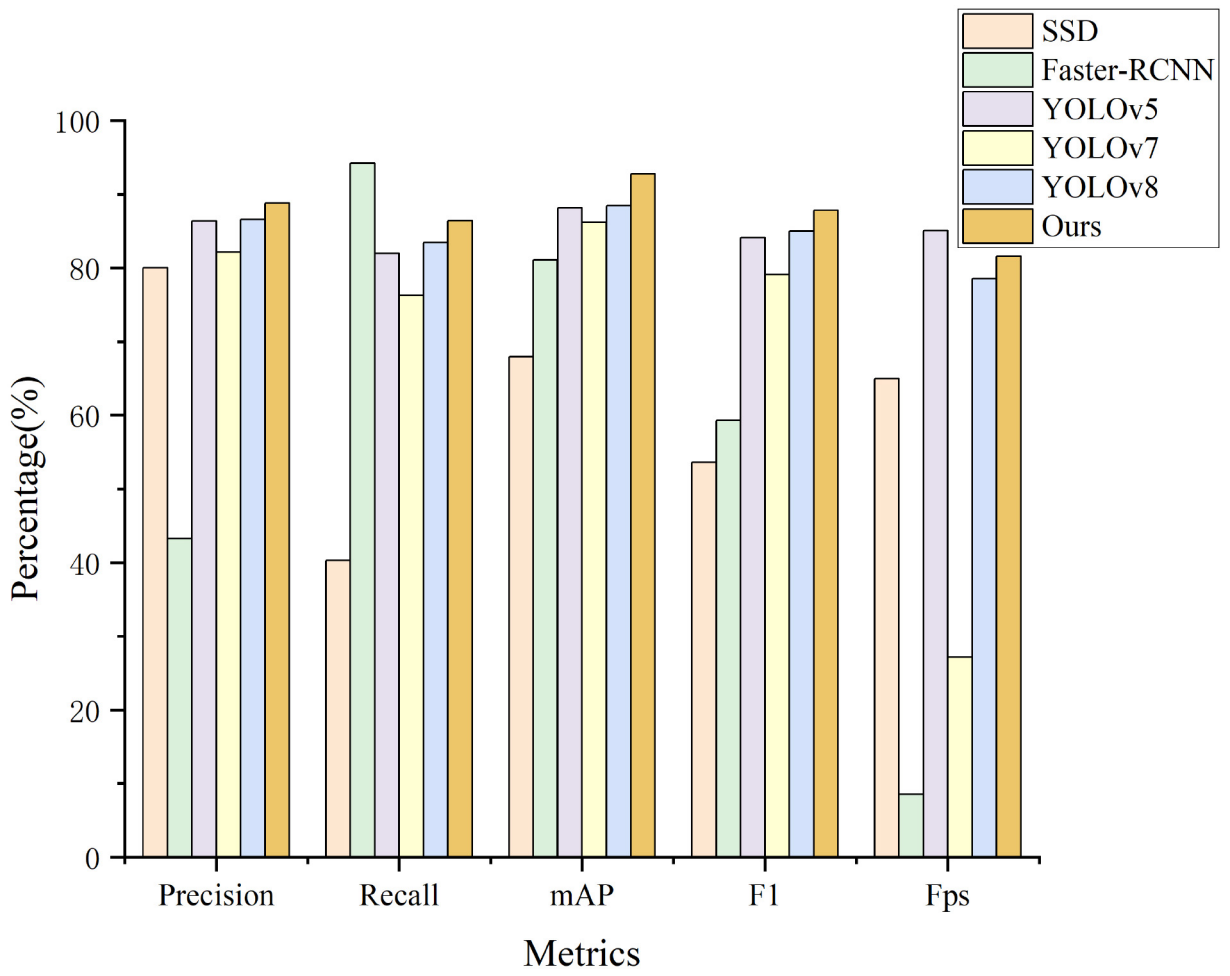
Precision, recall, mAP, F1, and FPS results from comparison experiments with object detection models.

**Figure 9:**
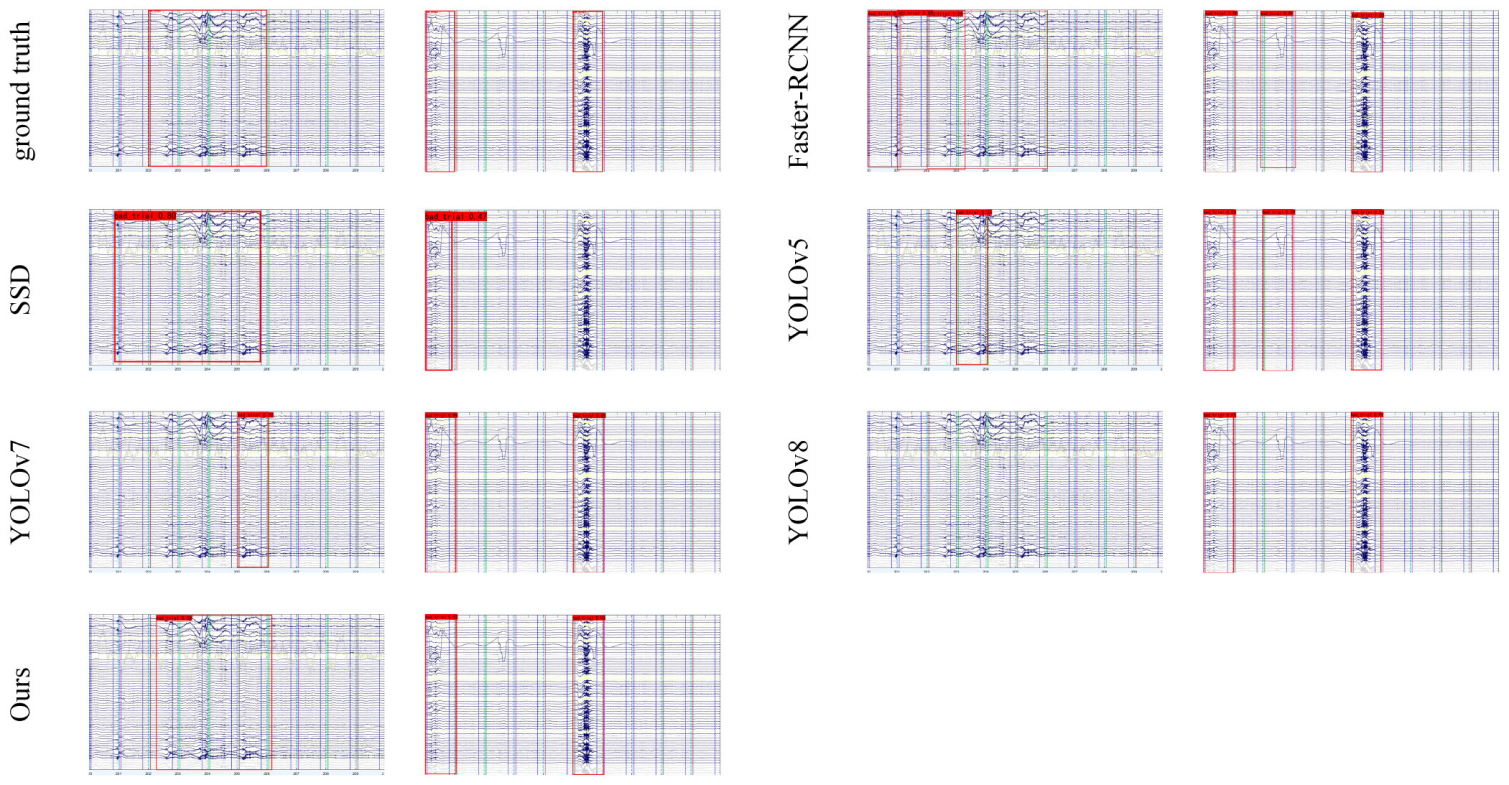
Representative comparison of different models for EEG bad trial detection: SSD, Faster-RCNN, YOLOv5, YOLOv7, YOLOv8, and our model.

For object detection models, we selected SSD, Faster-RCNN, YOLOv5, YOLOv7, and YOLOv8. We evaluated model performance using precision, recall, mAP, F1 score, and processing speed (Fps). The results are presented in Table 4 and visualized in Figure 8. Figure 9 displays comparative detection results with confidence scores.

**Table 4:** Comparison with object detection models on our dataset.

**Method**	**Pre (%)**	**Rec (%)**	**mAP (%)**	**F1 (%)**	**Fps**	**Params**	**GFLOPs**
SSD	80.00	40.29	67.99	53.59	64.90	26M	31
Faster-RCNN	42.23	**94.24**	81.08	58.32	8.50	41M	180
YOLOv5s	86.36	82.01	88.16	84.13	**85.04**	**7.2M**	**16.5**
YOLOv7	82.17	76.36	86.19	79.10	27.20	36.9M	104.7
YOLOv8s	86.57	83.45	88.46	84.98	78.54	11.2M	28.6
Ours	**88.76**	86.89	**92.76**	**87.82**	81.62	11.0M	32.3

Bold values indicate the best performance for each metric.

Among the compared methods, Faster-RCNN achieved the highest recall of 94.24% due to its two-stage detection approach, which identifies more candidate regions. However, this also led to the lowest precision of 42.23% and slowest processing speed of 8.50 Fps. SSD showed insufficient feature extraction capability for artifact segments, resulting in only 40.29% recall. YOLOv5, YOLOv7, and YOLOv8 exhibited more balanced performance across all metrics. YOLOBT outperformed all baseline models, achieving 88.76% precision, 86.89% recall, 92.76% mAP, and 87.82% F1 score while maintaining efficient processing at 81.62 Fps.

As illustrated in Figure 9, YOLOBT demonstrates superior detection capability across various challenging scenarios. In the left figure showing consecutive bad trials, YOLOBT maintained robust performance while other models exhibited both missed and false detections. In the right figure, some models incorrectly labeled trials with single electrode disturbances as bad trials, whereas YOLOBT avoided such misclassifications through CLAB's enhanced feature extraction.

We further compared YOLOBT with Autoreject (Jas et al., 2017), a representative traditional signal processing method, and two temporal deep learning methods: LSTM (Hochreiter and Schmidhuber, 1997) and EEGNet (Lawhern et al., 2018). For temporal methods, we implemented a sliding window approach where each trial was segmented into 2-s windows, each window was classified as artifact-contaminated or clean, and a trial was marked as bad if any window was classified as contaminated. Since these methods perform trial-level binary classification rather than object detection, we report precision, recall, and F1 score for comparison. Results are presented in Table 5.

**Table 5:** Comparison with traditional and temporal deep learning methods.

**Method**	**Pre (%)**	**Rec (%)**	**F1 (%)**
Autoreject	71.23	69.45	70.33
LSTM	47.52	84.67	60.88
EEGNet	43.18	86.21	57.56
Ours	**88.76**	**86.89**	**87.82**

Bold values indicate the best performance for each metric.

LSTM and EEGNet achieved high recall exceeding 84% but remarkably low precision below 48%, resulting in F1 scores of only 60.88 and 57.56%, respectively. This imbalance stems from their reliance on fixed thresholds applied uniformly across all trials regardless of overall signal quality, leading to excessive false positives when encountering minor artifacts that experts would tolerate in lower-quality recordings. Additionally, these temporal methods require fixed input dimensions determined by electrode configurations, necessitating retraining when applied to different montages or when channels are missing. Autoreject achieved more balanced performance with 71.23% precision, 69.45% recall, and 70.33% F1 score by incorporating automated threshold selection. However, its effectiveness remains limited by traditional signal processing constraints.

YOLOBT substantially outperformed all these methods by achieving the highest scores across all metrics. The key advantage lies in its ability to assess global signal quality through the GICM module and dynamically adjust artifact detection thresholds accordingly, precisely replicating the adaptive strategies employed by human experts. Furthermore, YOLOBT offers electrode-agnostic operation: it can process EEG visualizations from any recording system regardless of electrode count or montage, enhancing its applicability across diverse clinical and research settings.

### 4.6 Robustness analysis under different signal conditions

To further verify the stability and robustness of YOLOBT, we performed a stratified performance analysis across different recording conditions. Instead of using synthetic artifact injection—which could potentially compromise the integrity of the original expert annotations—we categorized the existing clinical test set into distinct artifact severity levels using quantitative signal metrics.

**Figure 10:**
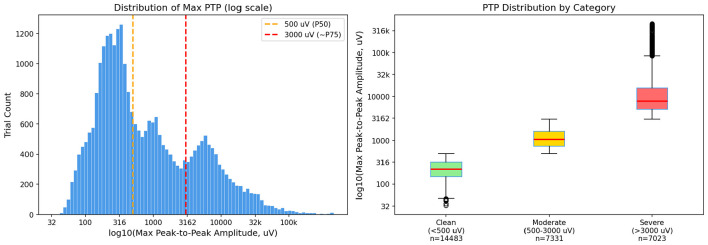
Quantitative characterization of artifact severity categories. **(Left)** Distribution of maximum peak-to-peak amplitude (Max PTP) across all trials on a logarithmic scale. Dashed lines indicate the category boundaries at 500 μV (median) and 3,000 μV (~75th percentile). **(Right)** Box plots showing the Max PTP distributions for each defined category, confirming clear separation between severity levels.

Specifically, we computed the maximum peak-to-peak amplitude (Max PTP) across all channels for each trial in our dataset. As shown in Figure 10, the distribution of Max PTP is heavily right-skewed, spanning from 32.65 to 453,768.38 μV (median = 493.53 μV, mean = 4,635.07 μV). Based on this distribution, we defined three artifact severity categories using the following quantitative criteria:

- Severe artifacts (Max PTP > 3,000 μV): trials exhibiting high-amplitude noise or intense muscle activity (EMG), accounting for 24.4% of all trials (median Max PTP = 7,744.4 μV, median Max STD = 1,857.2 μV).
- Moderate artifacts (500 μV < Max PTP ≤ 3,000 μV): trials with moderate noise levels or subtle waveform distortions, accounting for 25.4% of all trials (median Max PTP = 1,057.3 μV, median Max STD = 211.3 μV).
- Bad channel interference (Max PTP ≤ 500 μV with identified bad channels): trials that contain one or more identified bad channels within the recording segment, accounting for the remaining trials with relatively clean signals (median Max PTP = 219.9 μV, median Max STD = 38.2 μV).

The threshold of 3,000 μV corresponds approximately to the 75th percentile of the Max PTP distribution, above which artifacts are unambiguously severe. The threshold of 500 μV corresponds to the median of the distribution. These quantitative boundaries were further validated against expert annotations, confirming that the defined categories accurately reflect clinical artifact severity levels.

The model performance results under each condition, summarized in Table 6, demonstrate that YOLOBT maintains consistent performance across these quantitatively defined categories. The model achieves balanced Precision and Recall for both Severe and Moderate artifacts, with F1 scores of 85.16 and 86.93%, respectively. Even in trials containing bad channel interference, the model accurately identified bad trials with an F1 score of 87.64%. The absence of significant performance fluctuation across these categories confirms that YOLOBT is highly adaptive and can robustly handle the diverse signal complexities encountered in real-world clinical EEG data.

**Table 6:** Model performance under different artifact and signal conditions.

**Condition category**	**Pre (%)**	**Rec (%)**	**F1 (%)**
Severe artifacts (>3,000 μV)	84.92	85.41	85.16
Moderate artifacts (500–3,000 μV)	87.65	86.23	86.93
Bad channel interference ( ≤ 500 μV)	88.56	86.73	87.64
**Overall average**	**88.76**	**86.89**	**87.82**

Bold values indicate the best performance for each metric.

### 4.7 Comparison of bad trial detection for different signal qualities

**Figure 11:**
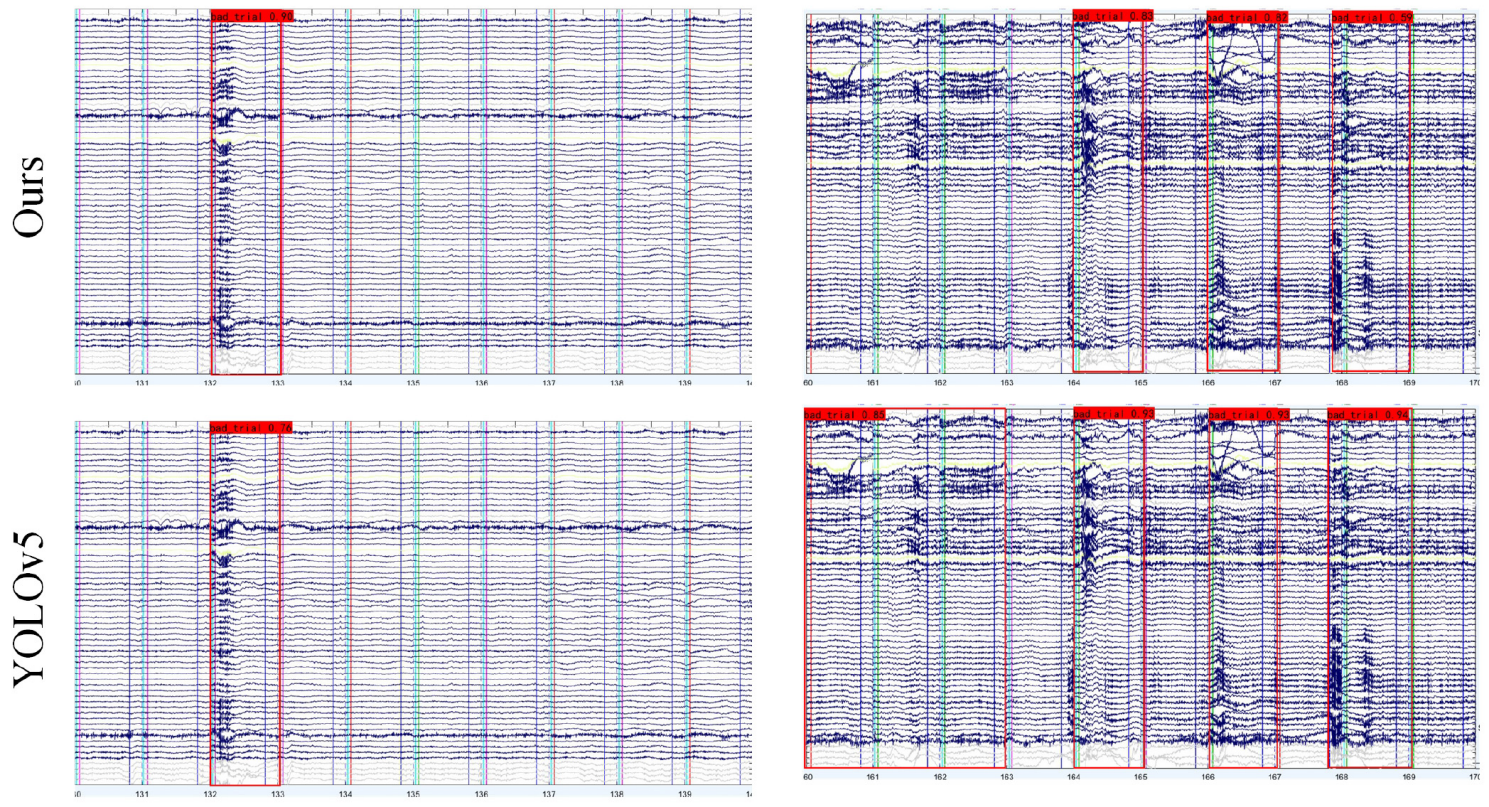
EEG bad trial detection results from two subjects with different signal qualities. **(Left)** An EEG image with better signal quality. **(Right)** An EEG image with poorer signal quality. The results demonstrate that our model dynamically adjusts artifact tolerance based on overall EEG signal quality.

To further illustrate the model's capability to dynamically adjust artifact detection based on signal quality, we compared detection results on two EEG images with different signal qualities against baseline YOLOv5 results, as shown in Figure 11. The left images exhibit significantly higher global signal quality than the right images.

In the high-quality signal case, YOLOBT identifies an artifactual trial with high confidence of 0.93, while YOLOv5 marks the same trial with lower confidence of 0.76. For the lower-quality signal, despite all trials containing slight disturbances and some having more severe artifacts than those in the high-quality image, YOLOBT correctly identifies only three trials as bad—matching expert annotations. Notably, the second trial in the lower-quality image, though containing artifacts similar to those in the high-quality image, is not flagged as bad by YOLOBT.

Furthermore, the three trials marked as bad in the lower-quality image contain more severe artifacts than the one in the high-quality image, yet YOLOBT assigns them lower confidence scores of 0.83, 0.82, and 0.59 compared to 0.93, 0.93, and 0.94. In contrast, YOLOv5 indiscriminately marks the first three trials as bad without apparent threshold adjustment. These results demonstrate that our proposed model effectively adjusts artifact tolerance based on overall signal quality, enabling dynamic bad trial judgment.

### 4.8 Heatmap visualization for YOLOBT

To further understand the decision-making mechanism of the YOLOBT model in bad trial detection tasks, we utilized Grad-CAM++ (Chattopadhay et al., 2018) to generate heatmaps visualizing the key regions of interest to the model. Through these heatmap analyses, we discovered that the model employs two primary decision-making patterns.

**Figure 12:**
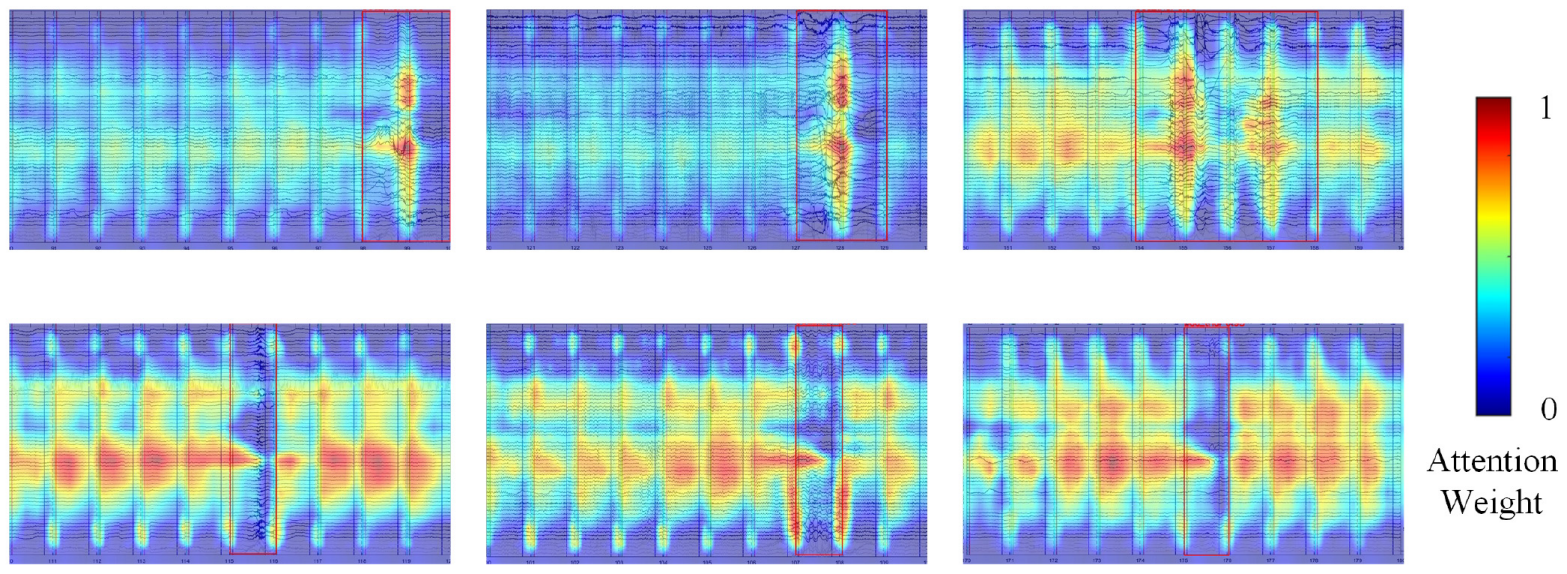
Attention heatmap visualization of YOLOBT using Grad-CAM++. Warmer colors (red/yellow) indicate regions of higher model attention, while cooler colors (blue) indicate lower attention. **(Top row)** When obvious artifacts are present, attention primarily focuses on artifact regions while also considering other channels within the same trial and corresponding positions in other trials. **(Bottom row)** In good quality signals, attention is widely distributed across artifact-free regions for global signal quality assessment.

When obvious artifacts appear in the EEG, the model allocates its primary attention to these artifact regions while also distributing auxiliary attention across two key dimensions: focusing on other channels within the same trial and on the same position in other trials. This dual comparative analysis, both within and across trials, enables the model to comprehensively evaluate the nature and severity of artifacts. As shown in the first row of Figure 12, the model's primary attention hotspots in Signal B concentrate on the obvious artifact region, while partial attention is allocated to other channel positions within the same trial, with attention distribution also present at corresponding positions in other trials. This multi-point attention allocation pattern indicates that the model does not evaluate artifacts in isolation when determining bad trials, but rather synthesizes the relative significance of artifacts through comparative analysis across channels and trials before deciding whether to mark a trial as bad.

In EEG images with overall better signal quality, the model exhibits distinctly different decision-making behavior. In these cases, the model does not directly focus on specific areas containing minor artifacts, but instead broadly distributes its attention across other trial regions, performing a global signal quality assessment. By focusing on high-quality trial regions to establish a signal quality baseline, the model can more precisely identify trials of relatively lower quality and accordingly lower its artifact tolerance threshold in high-quality signal environments. As shown in the second row of Figure 12, in samples with excellent signal quality, the model's attention displays a dispersed distribution pattern, with numerous hotspots distributed across artifact-free trial regions, confirming that the model is executing an evaluation strategy based on global quality standards rather than making isolated judgments based on single artifacts.

As a deep learning approach, YOLOBT learns discriminative features implicitly through end-to-end training rather than relying on explicitly predefined features. This paradigm is consistent with established practices in medical image analysis, where deep learning models achieve expert-level performance through learned representations. For instance, Esteva et al. (2017) demonstrated CNN-based skin cancer classification achieving dermatologist-level accuracy with model interpretation relying on visualization techniques, and Gulshan et al. (2016) developed deep learning for diabetic retinopathy detection without explicit feature engineering.

In our work, the Grad-CAM++ visualization provides interpretable insights into the decision-making basis of YOLOBT. The heatmap analysis reveals two distinct learned strategies that serve as implicit feature indicators for bad trial detection: (1) Multi-dimensional comparative analysis: When artifacts are present, the model simultaneously attends to artifact regions, other channels within the same trial, and corresponding positions in other trials. This attention pattern indicates that the model has learned to evaluate artifact severity through both cross-channel and cross-trial comparison rather than analyzing artifacts in isolation. (2) Global quality baseline establishment: In high-quality signal environments, the model broadly distributes attention across artifact-free regions instead of focusing on specific locations. This dispersed attention pattern suggests the model establishes a signal quality baseline from clean trials, enabling adaptive threshold adjustment based on overall data quality. These two complementary strategies mirror expert visual inspection processes, where researchers consider both local artifact characteristics and global signal context when making bad trial decisions. The interpretable attention patterns confirm that YOLOBT has learned clinically meaningful decision criteria through end-to-end training.

## 5 Discussion

Our findings demonstrate that YOLOBT significantly advances automated bad trial detection by achieving a precision of 88.76%, substantially outperforming baseline models and traditional approaches. The core innovation lies in the GICM module, which enables the framework to dynamically adjust artifact detection thresholds based on global signal quality. By mimicking the adaptive assessment strategies of human experts—who intuitively increase noise tolerance in lower-quality recordings to preserve statistical power—YOLOBT provides a context-aware solution that maintains analytical purity while maximizing data utility. This mechanism addresses a long-standing limitation in ERP preprocessing, where static thresholds often fail to capture the nuanced judgment required in diverse clinical scenarios.

The superiority of YOLOBT is further underscored by its ability to bridge the gap between automated efficiency and expert-level interpretability. Unlike temporal deep learning models (e.g., LSTM and EEGNet) that frequently suffer from low precision due to their reliance on local features, YOLOBT leverages computer vision to establish global quality baselines. Our heatmap analysis confirms that the model's decision-making—characterized by multi-dimensional comparative analysis across channels and trials—aligns closely with clinical visual inspection. Furthermore, the electrode-agnostic nature of this image-based approach ensures its scalability across different recording montages and research sites, facilitating the consistent preprocessing standards necessary for large-scale multi-site collaborations and meta-analyses.

While YOLOBT demonstrates high robustness, as evidenced by our stratified analysis of real-world clinical artifacts, we acknowledge certain limitations regarding its current generalizability. Our dataset originates from specific patient populations and a 64-channel system; thus, the subjective nature of “bad trial” definitions across different laboratories remains a challenge. We opted for a stratified evaluation of naturally occurring clinical artifacts rather than synthetic noise injection to ensure the model was tested against the true complexity of neurophysiological data. Future work will focus on integrating YOLOBT into real-time acquisition systems and extending its attention mechanisms to other EEG-based diagnostic tasks, such as seizure detection or sleep staging, ultimately establishing it as a standardized tool for automated neurophysiological quality control.

## 6 Conclusion

This paper proposed YOLOBT, a YOLO-based deep learning framework for automated bad trial detection in EEG-based ERP studies. By treating EEG signals as visualized waveform images, our approach aligns with expert visual inspection methods while enabling context-aware artifact detection. Three key modules were introduced: the Cross-Layer Attention Bottleneck (CLAB) for enhanced feature extraction, the Hierarchical Feature Guidance Module (HFGM) for multi-scale feature integration, and the Global Information Classification Module (GICM) for dynamic threshold adjustment based on signal quality. Experimental results demonstrated that YOLOBT achieved 88.76% precision, 86.89% recall, 92.76% mAP, and 87.82% F1 score, outperforming classical object detection models, traditional signal processing methods, and temporal deep learning approaches. Heatmap visualization confirmed that the model adaptively adjusts detection strategies based on signal quality, mirroring expert judgment processes. YOLOBT offers a practical solution for automating ERP preprocessing while maintaining the adaptive, context-aware assessment characteristic of expert manual inspection.

## Data Availability

The data analyzed in this study is subject to the following licenses/restrictions: the EEG dataset used in this study contains clinical patient data and is subject to medical privacy protection regulations, making it not publicly available. Data access is restricted to authorized researchers from collaborating institutions and requires institutional review board approval and data use agreements. Requests to access these datasets should be directed to CW, superwcm@163.com.
